# Real-Time 3D Tracking of Multi-Particle in the Wide-Field Illumination Based on Deep Learning

**DOI:** 10.3390/s24082583

**Published:** 2024-04-18

**Authors:** Xiao Luo, Jie Zhang, Handong Tan, Jiahao Jiang, Junda Li, Weijia Wen

**Affiliations:** 1 Department of Physics, The Hong Kong University of Science and Technology, Hong Kong 999077, China; xluoay@connect.ust.hk; 2 Advanced Materials Thrust, The Hong Kong University of Science and Technology, Guangzhou 511400, China; jzhang151@connect.hkust-gz.edu.cn (J.Z.); johnjhjiang@hkust-gz.edu.cn (J.J.); jli842@connect.hkust-gz.edu.cn (J.L.); 3 Department of Individualized Interdisciplinary Program (Advanced Materials), The Hong Kong University of Science and Technology, Hong Kong 999077, China; htanaf@connect.ust.hk

**Keywords:** particle tracking, wide-field microscopy, deep learning, image visualization

## Abstract

In diverse realms of research, such as holographic optical tweezer mechanical measurements, colloidal particle motion state examinations, cell tracking, and drug delivery, the localization and analysis of particle motion command paramount significance. Algorithms ranging from conventional numerical methods to advanced deep-learning networks mark substantial strides in the sphere of particle orientation analysis. However, the need for datasets has hindered the application of deep learning in particle tracking. In this work, we elucidated an efficacious methodology pivoted toward generating synthetic datasets conducive to this domain that resonates with robustness and precision when applied to real-world data of tracking 3D particles. We developed a 3D real-time particle positioning network based on the CenterNet network. After conducting experiments, our network has achieved a horizontal positioning error of 0.0478 μm and a z-axis positioning error of 0.1990 μm. It shows the capability to handle real-time tracking of particles, diverse in dimensions, near the focal plane with high precision. In addition, we have rendered all datasets cultivated during this investigation accessible.

## 1. Introduction

Particle tracking serves as an effective approach to measuring and scrutinizing the motion of tiny objects, ranging from single molecules to cells. It finds wide-ranging applications in various fields, such as colloid physics, medical treatments, biological science, pharmaceutical research, and cellular behavior studies. For instance, it can be applied to particle tracking in holographic optical tweezers to derive the Mean Squared Displacement (MSD) curve, calibrate the optical stiffness [1,2], or analyze the movements of self-propelled particles [3]. In the field of biological sciences, particle localization is a technique used to observe the invasion process of viruses into cells [4,5] and analyze the diffusion dynamics of nanoparticles near cell membranes [6]. Meanwhile, it is also possible to count bacterial colonies using particle localization [7]. When it comes to drug applications, the reliability of carriers can be assessed by conducting a direct tracking of these carriers [8].

Traditional numerical calculation methods have demonstrated notable effectiveness in the domain of particle tracking [9,10,11,12]. Research by Parthasarathy et al. [9] has revealed that the imaging particle’s intensity is radially symmetric around its center. Based on this principle, they designed a rapid and precise particle tracking algorithm capable of sub-pixel positioning accuracy. Similarly, Kashkanova, A.D. et al. [10] introduced an image transformation technique to accentuate features possessing a high degree of radial symmetry. However, traditional numerical calculations come with numerous drawbacks, such as heavy computational demands, the inability to track multi-particles simultaneously, and the requirement for pre-set parameters. To overcome these challenges, researchers have begun to merge deep learning with particle localization, developing several high-performing tracking algorithms. Using Mie scattering theory to fit holograms is a common method for obtaining the three-dimensional information of particles. However, this method involves high computation and can only calculate the information for one particle at a time. More details can be found in Supplementary Materials.

Common object detection networks encompass R-CNN [13], Mask-RCNN [14], YoLov1~YoLov7 [15,16], Fast re-OBJ [17], CenterNet [18], or Siamese network [19]. For example, Suljagic, H. and Bayraktar, E. et al. [19] innovatively proposed a similarity-based person re-id framework with higher accuracy, fewer ID switches, and false positive and negative rates, called SAT. Altman, L.E. and Grier, D.G. et al. [20,21] devised a 3D particle tracking network rooted in YoLo, capable of achieving high-precision particle localization. Shao, S. et al. [22] designed a convolutional neural network following the U-net architecture to facilitate particle localization. Additionally, Midtvedt, B. et al. [23] introduced a convolutional neural network called WAC-NET, which achieves high accuracy in calculating particle size and refractive index values using two orders of magnitude less data than the standard method.

Nearly every method encounters the challenge of non-existent datasets and the complexity of optical systems. To address this issue, we chose wide-field illumination instead of a well-coherent laser source and developed a simple and efficient method for generating datasets. Acquiring holograms using wide-field illumination offers several advantages over those obtained via single-wavelength irradiation. It is more straightforward to operate and capable of distinguishing between particles located above and below the focus, which is hard to realize using a single-wavelength laser source [24]. Meanwhile, a single-wavelength laser source can generate strong interference, which is why many researchers use a system based on a well-coherent laser source to do the tracking. Such a system can perform well when the observing environment is ideal. However, such properties also magnify the structural defects of devices. For example, if we want to track particles in microchannels (channel sizes ranging from several micrometers to hundreds of micrometers) to analyze the drug delivery process, Casimir force measurements, group behavior of multi-particles, the tiny structure of microchannels, and the defects inside will affect the accuracy of particle tracking by adding unnecessary interference noise to the background generated by a laser source, which is inevitable during the process and limits the tracking performance. However, wide-field illumination will significantly reduce background noise, expanding the application scenarios of our system.

Furthermore, datasets serve as integral components of network training. However, most relevant works neither detail the processing of datasets nor disclose the datasets. Hence, this paper presents a straightforward, efficient, and low-cost 3D multi-particle tracking system. We used artificial datasets to train networks and then applied such networks to real-world situations. We have provided a detailed description of the dataset creation process, which effectively fills a gap in this field regarding dataset creation.

Furthermore, experimental findings have confirmed that networks trained on synthetic datasets maintain their effectiveness when applied to real data. Obtaining a large-scale dataset with accurate and detailed annotations takes time and effort, although our proposed method is simple and provides an effective solution.

**Figure 1:**
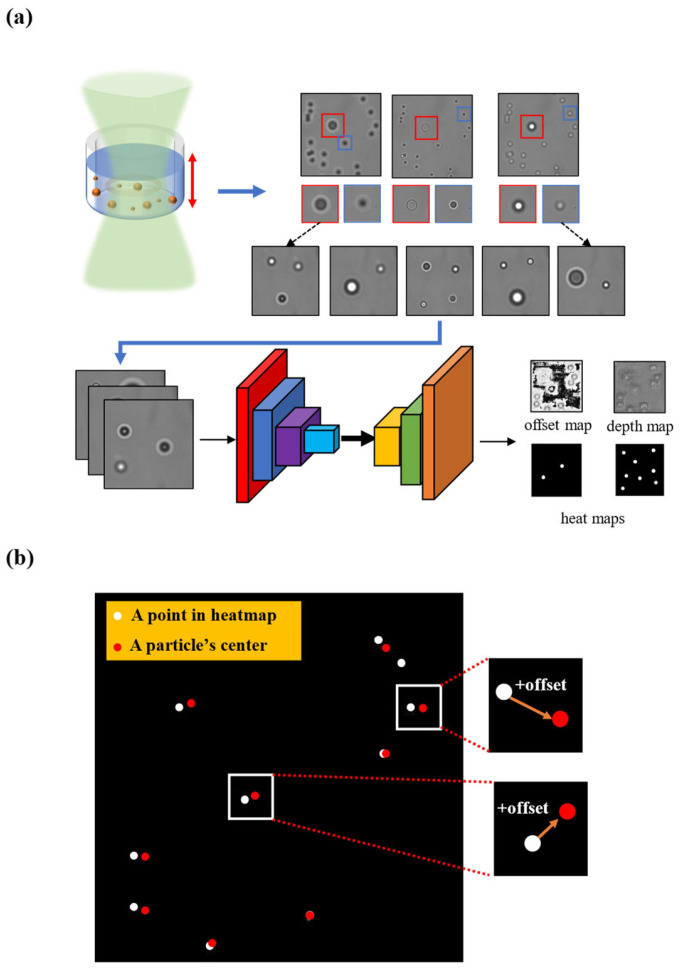
(**a**) Schematic representation of particle holograms analyzed using deep learning (Red square: Big particle; Blue square: Small particle). (**b**) Illustrated description of calculating particles’ centers.

Drawing inspiration from the CenterNet network, we approached particle localization as a unique application for keypoint detection and integrated the concept of a Feature Pyramid Network (FPN) [25] to propose a novel 3D localization network. Research findings demonstrate that this network has the capability to achieve real-time sub-micron-level planar positioning accuracy and axial positioning accuracy on both artificial and real datasets. Figure 1a depicts the workflow for analyzing particle holography using deep learning. The results from the trained network involve heat maps, offset maps, and depth maps. Utilizing heat maps and offset maps makes it feasible to calculate the horizontal position of particles in each image (Figure 1b).

## 2. Methodology

### 2.1. Production of Datasets

To facilitate the training of this network, we necessitated the preparation of a substantial quantity of data generated via the aforementioned optical tracking device. Manually labeling a significant volume of images and individually locating each particle proved challenging and time-consuming. The method we propose to make datasets solves this problem well.

A critical element of this process was the calibrating of each particle. Low labeling accuracy affects the predictive capabilities and the convergence of the network during training. In this experiment, we employed a high-precision stage to calibrate the particles at each location and capture the feature images. Given that the polystyrene beads with radii 1-μm and 2-μm were affixed to the cover glass, we could effectively discount movement around the coordinates attributable to Brownian motion. We scanned the beads from 10 μm below to above the focal plane along the z-axis, subdividing the distance into an average of 400 segments, each measuring 50 nm.

**Figure 2:**
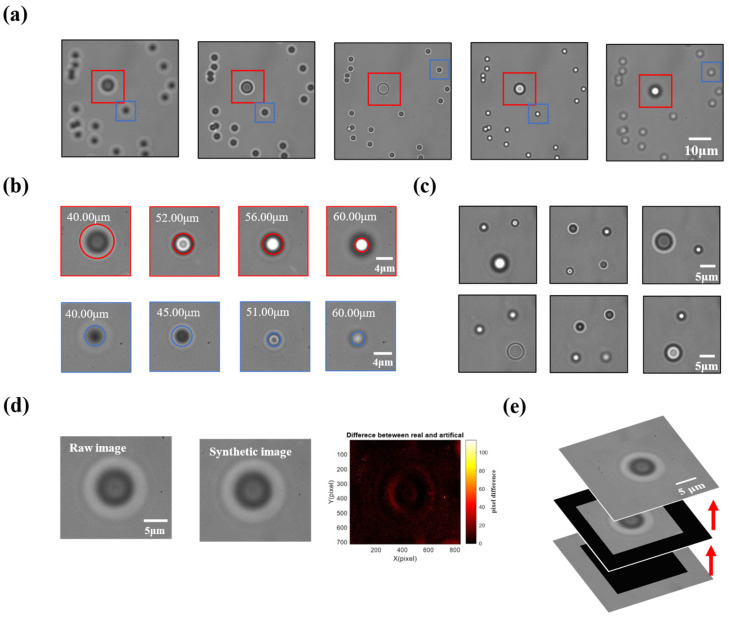
Schematic diagram of the process of creating artificial datasets. (**a**) Experimental images with different depths. The red and blue boxes indicate the small and large beads, respectively. (**b**) Feature images at different depths, with the red and blue boxes representing the small and large beads. The focal plane is at 50 μm. (**c**) Multiple particles with different depths in a synthetic image. (**d**) Comparison between artificial and original images, showing small differences characterized by randomization. (**e**) Schematic overview of the image fusion process.

We then captured the feature images of 1-μm and 2-μm beads at each position (Figure 2a). Following this procedure, we could calibrate 800 images of beads at differing positions. As shown in Figure 2b, as each feature image contained only one object, there were virtually no instances of inaccurate localization [9,10]. The images were randomly spliced into one image (Figure 2e) [26]. The difference between the synthesized and original images is random, which does not mislead the network to make incorrect judgments during the training process (Figure 2d). This approach enabled us to generate many bead images, each with well-calibrated data in the x, y, and z positions. The images in Figure 2c show some synthetic images.

### 2.2. Structure of the Network

**Figure 3:**
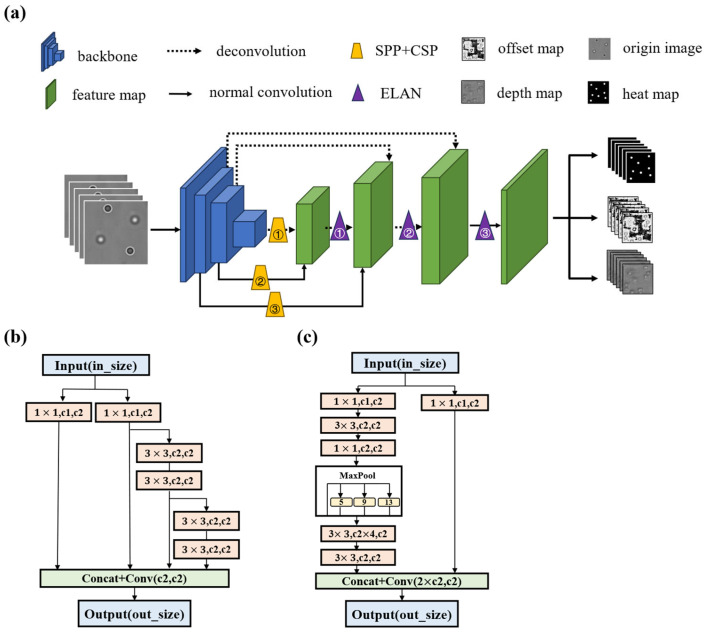
Schematic overview of the Estimator network. (**a**) Schematic diagram of network flow. A hologram is scaled to a standard size of 1024 pixels×1024 pixels before being fed into the network. (**b**,**c**) The schema of ELAN and SPP + CSP; 1×1 and 3×3 means the size of the convolution kernel; c1 and c2 mean channel amount of feature maps.

Deep learning plays a crucial role in the field of object detection. Not only have researchers proposed numerous excellent network architectures, but they have also introduced innovative algorithms such as Single Shot MultiBox Detector (SSD) [27] and Feature Pyramid Network (FPN). In Figure 3a, the flowchart of our network is presented. As our network is built upon the concept of the CenterNet network, for convenience, we refer to our network as CenterXFNet. Our network consists of three primary components: the Backbone; Neck; and Head modules.

For the Backbone network, we have selected Resnet50, a widely used and well-established choice among backbone networks. The Neck module primarily serves the purpose of information fusion. While a conventional CenterNet network performs only three standard upsampling operations during the Neck phase, our images have distinct characteristics with softly blurred edges in the particle pattern and a subtle difference between the images at varying depths. Softly blurred edges can make localization more difficult. Minor differences between images of different depths can cause the network’s depth prediction difficulties. These characteristics necessitate a better approach to blending our features. Our proposed Neck module draws concepts from the principles of FPN, as illustrated in Figure 3a. The Neck module mainly comprises several ELAN and SPP + CSP modules [16], as shown in Figure 3b,c. Table 1 and Table 2 show the relevant parameter settings in the Neck module. This methodology significantly enhances the informational density of the subsequent features. Moreover, this module can also be integrated into other network architectures, potentially improving their performance in object detection and localization tasks.

## 3. Experimental Methods

### 3.1. Sample Preparation

We chose 1-μm and 2-μm polystyrene beads to prepare the standard calibration solution. We centrifuged (1200 rpm) the beads and transferred them into a sodium chloride solution (1 M). We proceeded by depositing 120 μL of this solution into the spacer, which was then placed in an oven (70 °C) overnight. This process was designed to ensure the beads would adhere to the surface of the cover glass. Following this, we thoroughly and gently cleaned the cover glass to eliminate any beads that had not bonded. Finally, we filled the spacer with deionized water and carefully sealed it.

### 3.2. Tracking System Setup

**Figure 4:**
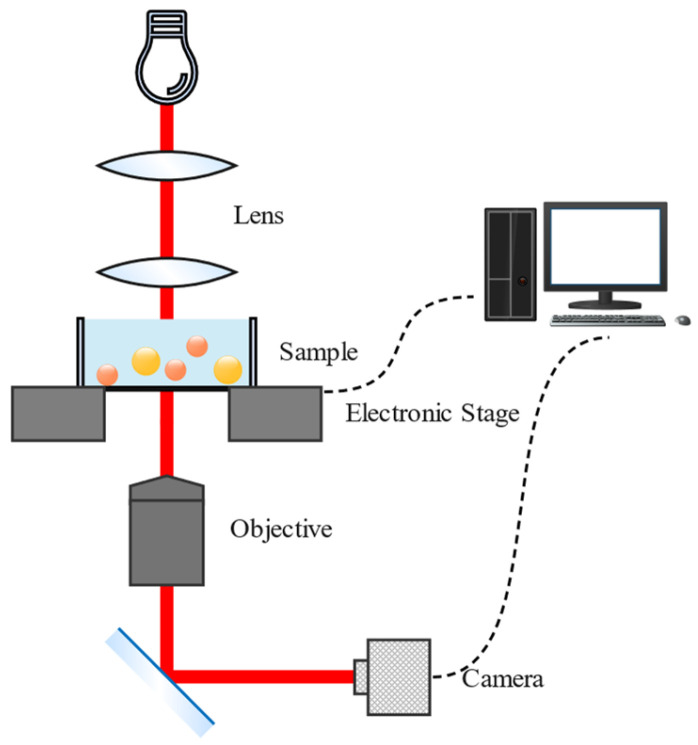
The optical path of the tracking system.

We chose wide-field illumination (Figure 4) to generate the dataset. A nano piezo z-stage (MCL) used in conjunction with the x−y stage (Nikon) calibrates the spatial coordinates. Given the precise accuracy requirement along the z-axis, each step measures 50 nm along this axis and 1 μm in the x−y plane. Movement is regulated by a PC-controlled stage, and a camera (Basler acA5472-17μmMED) captures several images at each position for calibration. Then, the video is processed by a PC to track multi-particle 3D positions in real time. The tracking software used in the experiments is self-developed.

## 4. Training and Evaluation

We created a dataset comprising 12,000 images to train the network, utilizing our innovative data production method. This training dataset consisted of 8400 images, while the evaluation dataset contained 3600 images. This dataset included two distinct types of particles with radii measuring 1 μm and 2 μm, positioned along the *z*-axis within a range of +10 μm to −10 μm from the focal plane. The image size varied between 950 and 1050 pixels, with each pixel approximately corresponding to a scale of 23.3 nm.

We utilized two types of test datasets: artificial; and real datasets. The artificial dataset comprised 3352 targets generated using the same method as the training dataset. On the other hand, the real dataset consisted of 2976 objects captured by the microscope during the experiment. For further training details, please refer to the Supplementary Materials.

## 5. Results

### 5.1. Data Tracking Analysis

**Figure 5:**
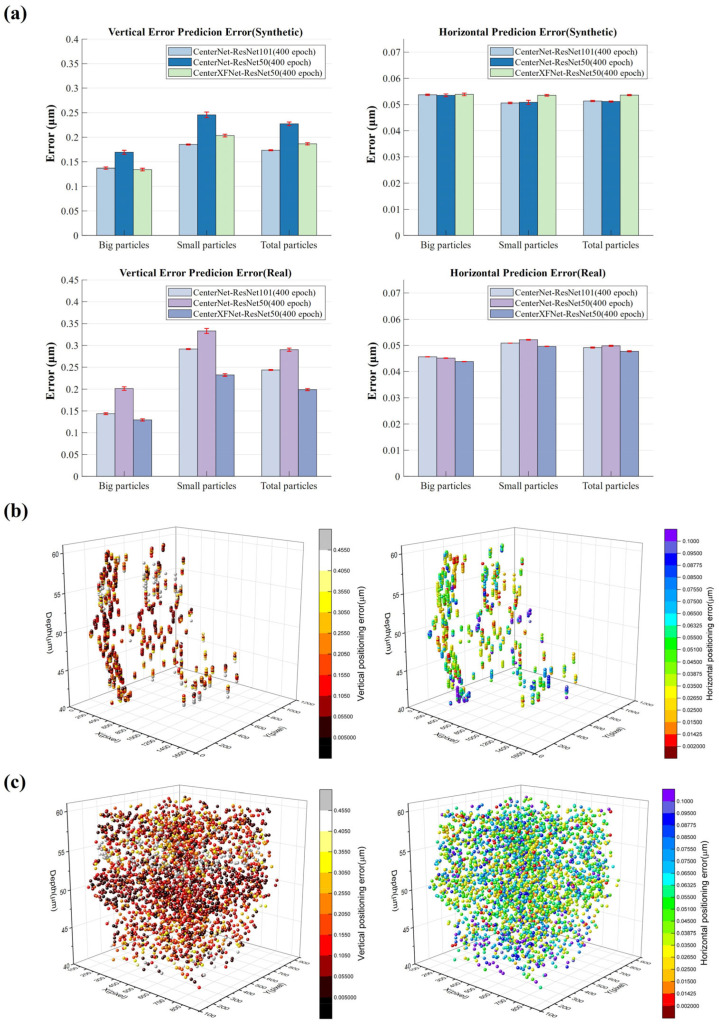
(**a**) The absolute error of horizontal and vertical positions in artificial data and real data. The red line indicates the standard deviation of multiple calculations. Horizontal and vertical error distributions in (**b**) real and (**c**) synthetic test datasets, respectively.

We evaluated the performance of our network using both artificial and real test datasets, and the corresponding results are presented in Figure 5. All three networks can achieve sub-micron-level positioning accuracy, whether on artificial or natural datasets. Specifically, the CenterNet network outperforms the CenterXFNet network on the artificial dataset, with the best horizontal and vertical positioning accuracies recorded at 0.0512 μm and 0.1740 μm, respectively. Meanwhile, the CenterXFNet-ResNet50 network exhibits significantly superior positioning capability on the real dataset compared to the other two networks, with the best horizontal and vertical positioning accuracies measured at 0.0478 μm and 0.1990 μm, respectively (Figure 5a). There are substantial disparities between natural and artificial data, which accounts for the differing positioning performance of the networks across the two datasets. The exceptional performance of the CenterXFNet on the real dataset suggests that, in contrast with the traditional CenterNet network, our proposed network has assimilated more genuinely advantageous features for particle positioning during the training process rather than merely focusing on the ineffectual features present in the artificial dataset.

Figure 5a illustrates particle size’s significant impact on positioning accuracy, particularly in the vertical direction. The network exhibits higher prediction accuracy for larger particles than small particles. This result is logical, as achieving precise positioning for small objects is a significant challenge in object detection.

Figure 5b,c shows the distribution of three-dimensional horizontal and vertical errors in the natural and synthetic test datasets. Additionally, it is worth emphasizing that their depth does not significantly affect the accuracy of predicting particles. In most research papers, obtaining depth information for particles above or below the focal plane is challenging, as the particle patterns observed in other experiments show no significant differences. However, the wide-field illumination technique we employed enables us to observe the differences between these particle patterns easily.

**Figure 6:**
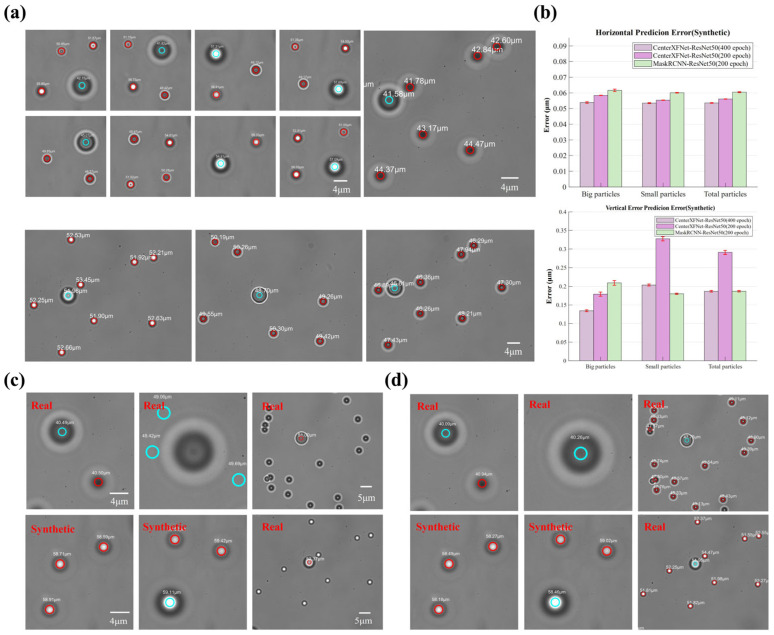
(**a**) The location result of CenterXFNet-ResNet50. Smaller images are synthetic images, and bigger images are experimental images. (**b**) Comparative results of CenterXFNet and Mask-RCNN. Localization results on real and synthetic datasets based on (**c**) Mask-RCNN-ResNet50 (200 epochs) and (**d**) CenterXFNet-ResNet50 (200 epochs), respectively. (Red circle: Small particle; Blue circle: Big particle).

Figure 6a displays the performance of CenterXFNet-Resnet50. This network demonstrates outstanding positioning capability in both artificial and natural images. The particle tracking video can be found in the referenced document (Video S1) for a more comprehensive understanding.

We also compared the CenterXFNet network with the Mask-RCNN network. We trained the CenterXFNet network and the Mask-RCNN network for 200 epochs separately. Figure 6b shows the comparative results of the two networks on the artificial dataset. Mask-RCNN demonstrates higher vertical prediction accuracy than the CenterXFNet network. However, the horizontal positioning capability is the opposite (Figure 6b). However, Mask-RCNN’s positioning ability on the real dataset is so poor that we cannot evaluate the network’s positioning ability on the real dataset (Figure 6c,d). We believe that similar to the traditional CenterNet, Mask-RCNN has been misled by irrelevant information in the artificial dataset during the learning process and has failed to learn the correct features. This further indicates that the CenterXFNet we proposed has superior feature learning capabilities. Additionally, the Mask-RCNN network makes it hard to meet real-time detection requirements.

To verify the reliability and reproducibility of our network, we have conducted a series of experiments (Table 3 and Table 4). In the horizontal direction, the positioning performance of the CenterXFNet network (without ELAN) shows better results (Table 3). However, the CenterXFNet network demonstrates more accurate localization capabilities in the vertical direction, especially for big particles (Table 4). The SPP + CSP and ELAN modules play different roles in predicting the three-dimensional information of beads. The ELAN module plays a very positive role in predicting the horizontal position of beads. However, the SPP + CSP and ELAN modules are essential for *z*-axis prediction. In our application, we value inference ability more in the vertical direction, so we chose to combine two modules.

### 5.2. Real-Time Tracking

**Figure 7:**
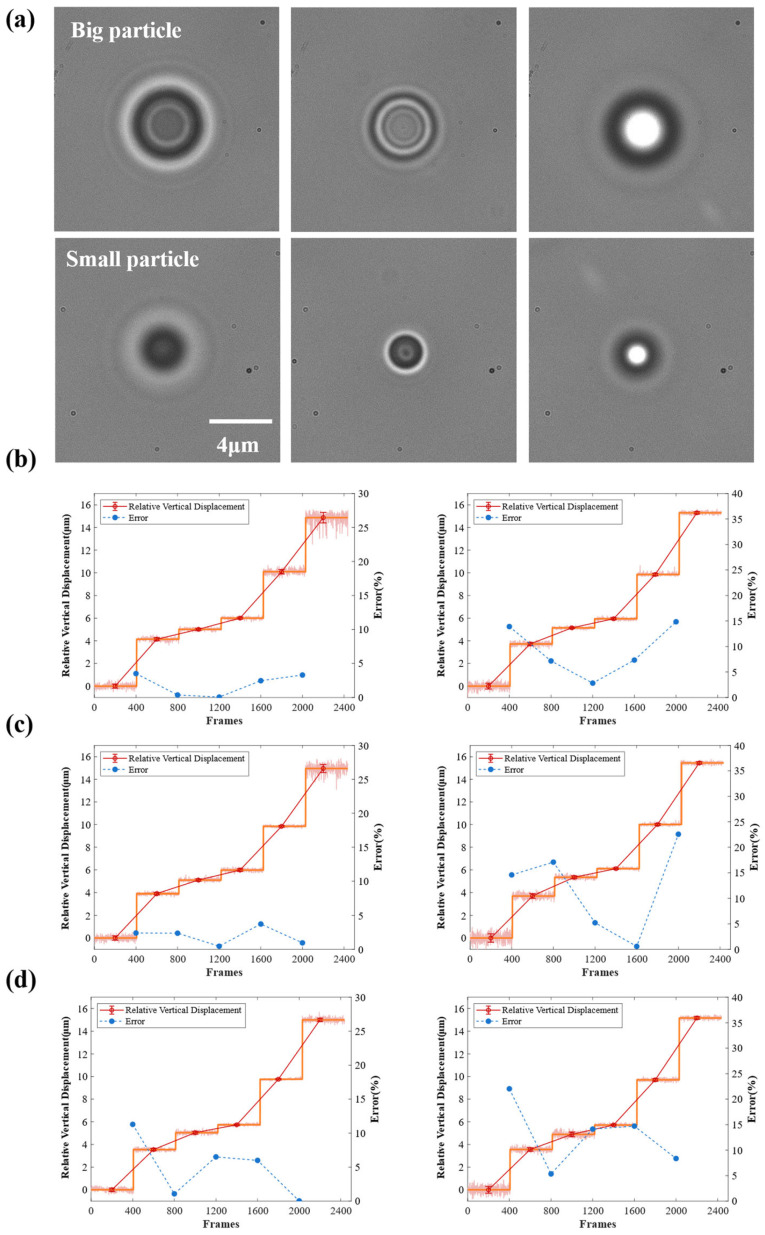
(**a**) Hologram of a big particle (4-μm PS bead) and small particle (2-μm PS bead). The depth positions of the particles are in order from left to right, from the above to the beneath of the focal plane. Real-time tracking and the tracking data distribution along the *z*-axis for (**b**) CenterXFNet-ResNet50, (**c**) CenterXFNet-ResNet101, and (**d**) CenterXFNet-DLA34, respectively. The left column shows the tracking results for the big particle. The right column is the tracking result of the small particle.

We implemented the trained network for real-time tracking and compared its performance on both large and small particles (Figure 7). To test the tracking system’s performance, the high-density particle mixture solution was allowed to settle within spacers for an adequate amount of time, causing the particles to deposit near the bottom of the cover glass and then stick to the cover glass surface. In this way, it can be considered that the unique displacement of the stage is the same as that of the beads, excluding the influence of the Brownian motion of the bead itself. We subsequently manipulated the electronic stage to different heights at −5 µm, −1 µm, 0 µm, 1 µm, 5 µm, and 10 µm (relative to the focal plane) and conducted real-time tracking to assess the system’s reliability by comparing the tracking results along the *z*-axis and the movements of the stage.

Figure 7b–d shows the tracking results of three different networks. It is pretty evident that the CenterXFNet-ResNet50 network is the best at what it achieves, and most of the relative tracking data of bead movements fall within a range of less than 10% of the bead size itself, spanning from −5 µm to 10 µm. We need to note that in the extensive particle real-time tracking experiments, the particle prediction results fluctuated because of the occurrence of particle collisions. In most cases, the networks with our proposed structure added in the Neck phase showed better predictions than the original networks. Comparison results can be found in Figures S3 and S4.

## 6. Conclusions

In our network, we incorporate the concept of key point detection, achieving sub-micron precision in both horizontal position and vertical depth positioning. This network is designed as an end-to-end system with a simplified architecture, which facilitates debugging and enhancement. We propose a method for dataset generation that is both rapid and straightforward. Meanwhile, we propose using wide-field illumination instead of single-wavelength illumination, which reduces costs and decreases noise, expanding the applicability of our method. Importantly, we demonstrate that networks trained on these synthetically produced datasets are also effective when applied to real data and real-time tracking, which can give guidance in colloid biophysics and drug delivery research in medical treatment.

Utilizing the Mie scattering theorem to obtain three-dimensional position information of particles presents several drawbacks, including significant computational complexity, the inability to simultaneously track multiple particles, and the necessity for predefined parameters [28]. In contrast, our proposed algorithm enables real-time multi-particle tracking without prior knowledge. The successful implementation of our algorithm implies the necessity to reassess the application of the Mie scattering theorem. Furthermore, it inspires us to explore the potential integration of deep learning with other fields. For instance, applying the Rayleigh–Sommerfeld formula for light field reconstruction requires high computational complexity and requires us to filter the optimal target points artificially [28]. This encourages us to investigate whether deep-learning methods can effectively address these limitations and expand their application scope.

At present, our network is capable of detecting spherical particles. However, with the appropriate training, the detector can accurately position particles of other shapes, including rods, cones, or irregular shapes.

A large-scale dataset with accurate and detailed calibration information is indispensable to address the above-mentioned issue. However, obtaining such image data is very troublesome and challenging. In the absence of a large-scale dataset, we should consider employing unsupervised learning methods to broaden the applicability of the locator to various scenarios. The emergence of large-scale networks such as CLIP [29] also opens the possibility of utilizing one-shot learning methods and few-shot learning methods. Our network’s simplicity allows for more optimization potential to achieve higher precision in particle positioning. Moreover, further simplifying and deploying the network to an Edge Computing Device could significantly enhance its efficiency.

## Figures and Tables

**Table 1:** Detailed parameters of SPP + CSP module (CenterXFNet-Resnet50).

IDX	C1	C2	In_Size	Out_Size	In_Channel	Out_Channel
SPP + CSP 1	2048	256	(32,32)	(32,32)	2048	256
SPP + CSP 2	1024	256	(64,64)	(64,64)	1024	256
SPP + CSP 3	512	128	(128,128)	(128,128)	512	128

**Table 2:** Detailed parameters of ELAN module (CenterXFNet-Resnet50).

IDX	C1	C2	In_Size	Out_Size	In_Channel	Out_Channel
ELAN 1	256	512	(64,64)	(64,64)	256	256
ELAN 2	128	256	(128,128)	(128,128)	128	128
ELAN 3	64	128	(256,256)	(256,256)	64	64

**Table 3:** Horizontal localization accuracy on different training parameters and network structures.

Input_Size	Epoch	SPP + CSP	ELAN	Error (Big) (μm)	Error (Small) (μm)	Error (Total) (μm)
(1024,1024)	400	P	P	0.0438 ± 0.43 × 10^−3^	0.0496 ± 0.18 × 10^−3^	0.0478 ± 0.79 × 10^−3^
(512,512)	400	P	P	0.0468 ± 0.41 × 10^−3^	0.0531 ± 0.44 × 10^−3^	0.0510 ± 0.25 × 10^−3^
(1024,1024)	200	P	P	0.0581 ± 0.36 × 10^−3^	0.0549 ± 0.33 × 10^−3^	0.0557 ± 0.20 × 10^−3^
(1024,1024)	400	O ^1^	P	**0.0428 ± 0.36 × 10^−3^**	0.0478 ± 0.39 × 10^−3^	0.0462 ± 0.31 × 10^−3^
(1024,1024)	400	P	O ^2^	0.0432 ± 0.31 × 10^−3^	**0.0464 ± 0.36 × 10^−3^**	**0.0454 ± 0.25 × 10^−3^**

^1^ Replace SPP + CSP block with a Conv2d + BN + ReLu block. ^2^ Replace ELAN block with a Conv2d + BN + ReLu block.

**Table 4:** Vertical localization accuracy on different training parameters and network structures.

Input_Size	Epoch	SPP + CSP	ELAN	Error (Big) (μm)	Error (Small) (μm)	Error (Total) (μm)
(1024,1024)	400	P	P	**0.1293 ± 0.16 × 10^−2^**	0.2322 ± 0.62 × 10^−2^	**0.1988 ± 0.23 × 10^−3^**
(512,512)	400	P	P	0.2134 ± 0.32 × 10^−2^	0.4332 ± 0.14 × 10^−1^	0.3599 ± 0.10 × 10^−1^
(1024,1024)	200	P	P	0.1756 ± 0.44 × 10^−2^	0.3247 ± 0.77 × 10^−2^	0.2880 ± 0.69 × 10^−2^
(1024,1024)	400	O	P	0.1764 ± 0.29 × 10^−2^	**0.2154 ± 0.50 × 10^−2^**	0.2029 ± 0.42 × 10^−2^
(1024,1024)	400	P	O	0.1543 ± 0.38 × 10^−2^	0.2411 ± 0.64 × 10^−2^	0.2135 ± 0.48 × 10^−2^

## Data Availability

The Software and Dataset can be downloaded at https://github.com/Jeacy22/Tracking-3D-particles/tree/master (accessed on 16 April 2024).

## Supplementary Materials

The following supporting information can be downloaded at https://www.mdpi.com/article/10.3390/s24082583/s1, Note S1: Mie scattering theory; Note S2: Evaluation Metrics; Table S1: Training parameter setting (CenterXFNet-ResNet50); Figure S1: Statistics of the number of particles at different depths in the data set; Figure S2: Statistical graph of the number of particles of different sizes in the dataset; Figure S3: Comparison results of DLA34; Figure S4: Comparison results of ResNet101; Video S1: Multi-particle in Real Time.

## References

[1] Pesce G., Jones P.H., Maragò O.M., Volpe G. (2020). Optical tweezers: Theory and practice. Eur. Phys. J. Plus.

[2] Gieseler J., Gomez-Solano J.R., Magazzù A., Pérez Castillo I., Pérez García L., Gironella-Torrent M., Viader-Godoy X., Ritort F., Pesce G., Arzola A.V. (2021). Optical tweezers—From calibration to applications: A tutorial. Adv. Opt. Photonics.

[3] Novotný F., Wang H., Pumera M. (2020). Nanorobots: Machines squeezed between molecular motors and micromotors. Chem.

[4] Ma Y., Wang X., Liu H., Wei L., Xiao L. (2019). Recent advances in optical microscopic methods for single-particle tracking in biological samples. Anal. Bioanal. Chem..

[5] Liu S.-L., Wang Z.-G., Xie H.-Y., Liu A.-A., Lamb D.C., Pang D.-W. (2020). Single-virus tracking: From imaging methodologies to virological applications. Chem. Rev..

[6] Ye Z., Wei L., Zeng X., Weng R., Shi X., Wang N., Chen L., Xiao L. (2017). Background-free imaging of a viral capsid proteins coated anisotropic nanoparticle on a living cell membrane with dark-field optical microscopy. Anal. Chem..

[7] Zhang B., Zhou Z., Cao W., Qi X., Xu C., Wen W. (2022). A new few-shot learning method of bacterial colony counting based on the edge computing device. Biology.

[8] Caputo F., Clogston J., Calzolai L., Rösslein M., Prina-Mello A. (2019). Measuring particle size distribution of nanoparticle enabled Medicinal Products, the joint view of EUNCL and NCI-NCL. A step by step approach combining orthogonal measurements with increasing complexity. J. Control. Release.

[9] Parthasarathy R. (2012). Rapid, accurate particle tracking by calculation of radial symmetry centers. Nat. Methods.

[10] Kashkanova A.D., Shkarin A.B., Mahmoodabadi R.G., Blessing M., Tuna Y., Gemeinhardt A., Sandoghdar V. (2021). Precision single-particle localization using radial variance transform. Opt. Express.

[11] Yuen H., Princen J., Illingworth J., Kittler J. (1990). Comparative study of hough transform methods for circle finding. Image Vis. Comput..

[12] Flewellen J.L., Minoughan S., Garcia I.L., Tolar P. (2022). Digital Holography-based 3D particle localisation for single molecule tweezer techniques. Biophys. J..

[13] Girshick R., Donahue J., Darrell T., Malik J. Rich feature hierarchies for accurate object detection and semantic segmentation. Proceedings of the 2014 IEEE Conference on Computer Vision and Pattern Recognition.

[14] He K., Gkioxari G., Dollár P., Girshick R. (2018). Mask R-CNN. arXiv.

[15] Yasmine G., Maha G., Hicham M. Overview of single-stage object detection models: From YOLOV1 to Yolov7. Proceedings of the 2023 International Wireless Communications and Mobile Computing (IWCMC).

[16] Wang C.-Y., Bochkovskiy A., Liao H.-Y.M. Yolov7: Trainable bag-of-freebies sets new state-of-the-art for real-time object detectors. Proceedings of the 2023 IEEE/CVF Conference on Computer Vision and Pattern Recognition (CVPR).

[17] Bayraktar E., Wang Y., DelBue A. (2022). Fast re-OBJ: Real-time object re-identification in rigid scenes. Mach. Vis. Appl..

[18] Zhou X., Wang D., Krähenbühl P. (2019). Objects as Points. arXiv.

[19] Suljagic H., Bayraktar E., Celebi N. (2022). Similarity based person re-identification for multi-object tracking using Deep Siamese network. Neural Comput. Appl..

[20] Altman L.E., Grier D.G. (2020). Catch: Characterizing and tracking colloids holographically using deep neural networks. J. Phys. Chem. B.

[21] Altman L.E., Grier D.G. (2023). Machine learning enables precise holographic characterization of colloidal materials in real time. Soft Matter.

[22] Shao S., Mallery K., Kumar S.S., Hong J. (2020). Machine learning holography for 3D particle field imaging. Opt. Express.

[23] Midtvedt B., Olsén E., Eklund F., Höök F., Adiels C.B., Volpe G., Midtvedt D. (2021). Fast and accurate nanoparticle characterization using deep-learning-enhanced off-axis holography. ACS Nano.

[24] Bohren C.F., Huffman D.R. (1983). Absorption and Scattering of Light by Small Particles.

[25] Lin T.-Y., Dollár P., Girshick R., He K., Hariharan B., Belongie S. (2017). Feature Pyramid Networks for Object Detection. arXiv.

[26] Pérez P., Gangnet M., Blake A. (2003). Poisson image editing. ACM SIGGRAPH 2003 Papers.

[27] Liu W., Anguelov D., Erhan D., Szegedy C., Reed S., Fu C.-Y., Berg A.C. (2016). SSD: Single Shot Multibox Detector. arXiv.

[28] Cheong F.C., Krishnatreya B.J., Grier D.G. (2010). Strategies for three-dimensional particle tracking with holographic video microscopy. Opt. Express.

[29] Wang S., Duan Y., Ding H., Tan Y.-P., Yap K.-H., Yuan J. Learning transferable human-object interaction detector with natural language supervision. Proceedings of the 2022 IEEE/CVF Conference on Computer Vision and Pattern Recognition (CVPR).

