# Supplementary material for: Real-Time 3D Tracking of Multi-Particle in the Wide-Field Illumination Based on Deep Learning
Source: Sensors (Basel). 2024 Apr 18;24(8):2583. doi: 10.3390/s24082583 (PMC11054292; doi:10.3390/s24082583)
Supplement: Supplementary file 1 [file sensors-24-02583-s001.zip › Supporting Information.pdf]

Supporting Information

Supplementary Notes

<u>Supplementary Note 1- Mie scattering theory.</u>

Assuming a light beam polarized in the $\hat{\varepsilon_0}$ direction propagating along the $\hat{z}$, with an incident electric field frequency of ω and a wavelength of λ. Then, the plane wave equation for the incident light is given by,

$$\boldsymbol{E}_0(\boldsymbol{r}, z) = u_0(\boldsymbol{r}) e^{ikz} \hat{\varepsilon_0} \tag{1}$$

Where the wavenumber is $k = 2\pi n_m/\lambda$, a medium of refractive index is n_m. When a light beam is irradiated onto an isotropic small sphere, the expression of the reflected light field is described by,

$$\boldsymbol{E}_S(\boldsymbol{r}, z) = E_s(\boldsymbol{r}, z) \hat{\varepsilon}(\boldsymbol{r}, z) \tag{2}$$

The measured intensity at point r in the focal plane is due to the superposition of the incident and scattered waves. Thus, the normalized hologram of an isotropic sphere can be expressed by,

$$b(r) = \frac{|\boldsymbol{E}_0(\boldsymbol{r}, 0) + \boldsymbol{E}_S(\boldsymbol{r}, 0)|^2}{|u_0(\boldsymbol{r})|^2} \approx 1 + 2\Re\{E_R(\boldsymbol{r}, 0)\} + |E_R(\boldsymbol{r}, 0)|^2 \tag{3}$$

Where the reduced scattered field is $E_R(\boldsymbol{r}, 0) = E_s(\boldsymbol{r}, 0)/u_0(\boldsymbol{r})$.

According to the Mie theory, the scattered light field generated by a particle at the focal plane r_p can be given by,

$$\boldsymbol{E}_s(\boldsymbol{r}) = \boldsymbol{E}_0 e^{-ikz_p} f_s\left(k(\boldsymbol{r} - \boldsymbol{r_p})\right) \tag{4}$$

Then the normalized hologram can be expressed by,

$$b(r) = 1 + 2\Re\left\{e^{-ikz_p} f_s\left(k(r - r_p)\right) \cdot \hat{x}\right\} + \left|f_s\left(k(r - r_p)\right)\right|^2 \tag{5}$$

Lorenz-Mie theory describes the scattering function as an expansion,

$$f_s(k\boldsymbol{r}) = \sum_{n=1}^{n_c} \frac{i^n(2n+1)}{n(n+1)} \left(i\alpha_n \boldsymbol{N}_{e1n}^{(3)}(k\boldsymbol{r}) - \beta_n \boldsymbol{M}_{o1n}^{(3)}(k\boldsymbol{r})\right) \tag{6}$$

Equation (5), to fit the normalized particle hologram, can obtain the three-dimensional information of the particle. Using the Mie scattering to calculate the three-dimensional information of the small sphere requires calculating many parameters and only obtaining the three-dimensional information of a tiny sphere at a time.

Supplementary Note 2- Evaluation Metrics.

(1) Horizontal Position Error

The Horizontal Position Error is defined as:

$$XY_{error} = \sqrt{\left(x_g - x_p\right)^2 + \left(y_g - y_p\right)^2} \tag{7}$$

where x_g and y_g is the actual position in x-y plane, x_p and y_p is the predicted results.

(2) Vertical Position Error

The Vertical Position Error is defined as:

$$Z_{error} = \left|z_g - z_p\right| \tag{8}$$

where z_g is the actual position in the z-axis, z_p is the predicted results.

Supplementary Note 3- Training environment and parameter setting.

The network was trained for 400 epochs on a computer equipped with a GPU A40 (48GB) and a Platinum 8358P CPU @ 2.60GHz, which took approximately four days. Inference on a 4096*2168 pixels image takes 0.025s (40 FPS) on a computer equipped with a GPU 4090 (24GB) and an i7-11700@2.50GHz CPU, which fully meets real-time requirements.

Supplementary Figures

Supplementary Table S1

Tabel S1 Training parameter setting (CenterXFNet-ResNet50)

Parameters	Setting	Help
Unfreeze_Epoch	350	Training batches in unfrozen backbone condition
Freeze_Epoch	50	Training batches in frozen backbone condition
Batch_Size	8	Training batch size in unfrozen backbone condition
Freeze_Batch_Size	16	Training batch size in frozen backbone condition
Optimizer	Adam	betas = (0.9, 0.999), weight_decay = 1e-8
PreTrained	True	Using pre-trained weights
Input_Size	(1024,1024)	Image size for network input
Augmentation	Self-developed	Details can be found in the code and running logs
LR[1]	Self-developed	Details can be found in the code and running logs

[1] It is recommended that the provided weighting parameters be used. It may be necessary to increase the training batch appropriately in case of different learning rates.

Supplementary Figure S1

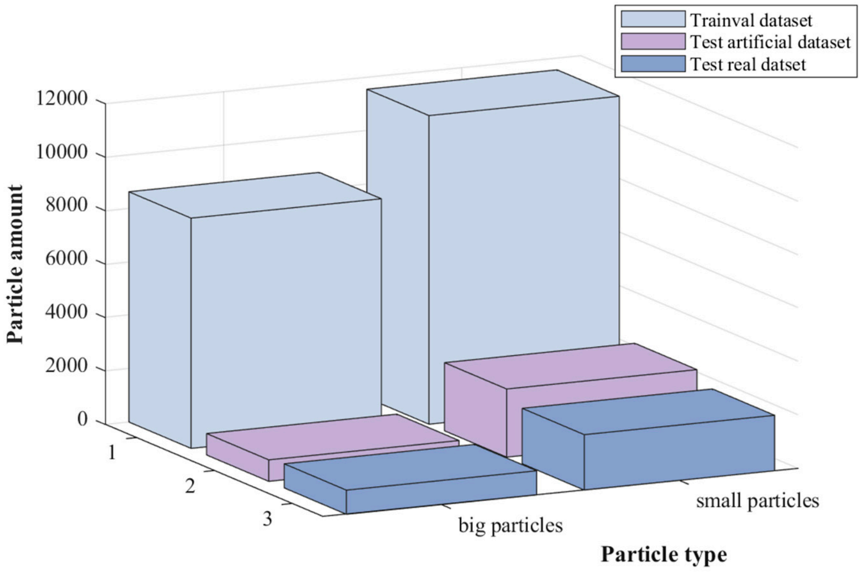

Figure S1 Statistics of the number of particles at different depths in the data set.

Supplementary Figure S2

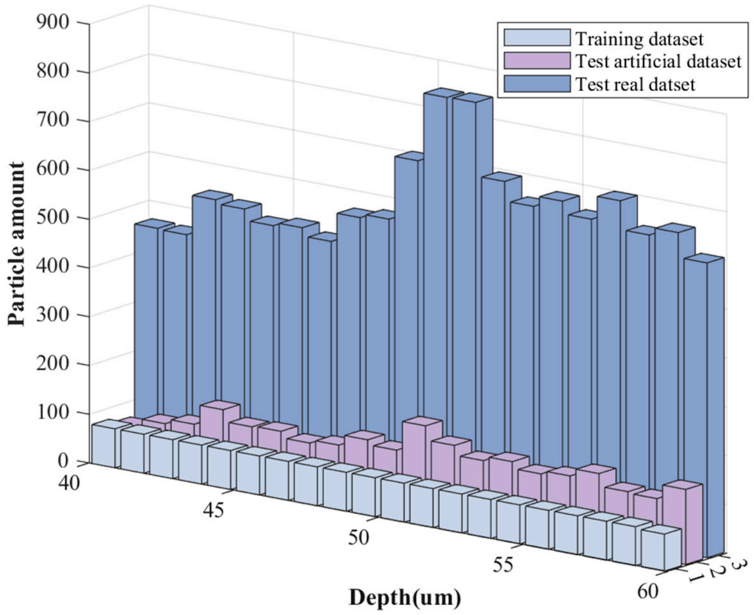

Figure S2 Statistical graph of the number of particles of different sizes in the dataset.

<u>Supplementary Figure S3</u>

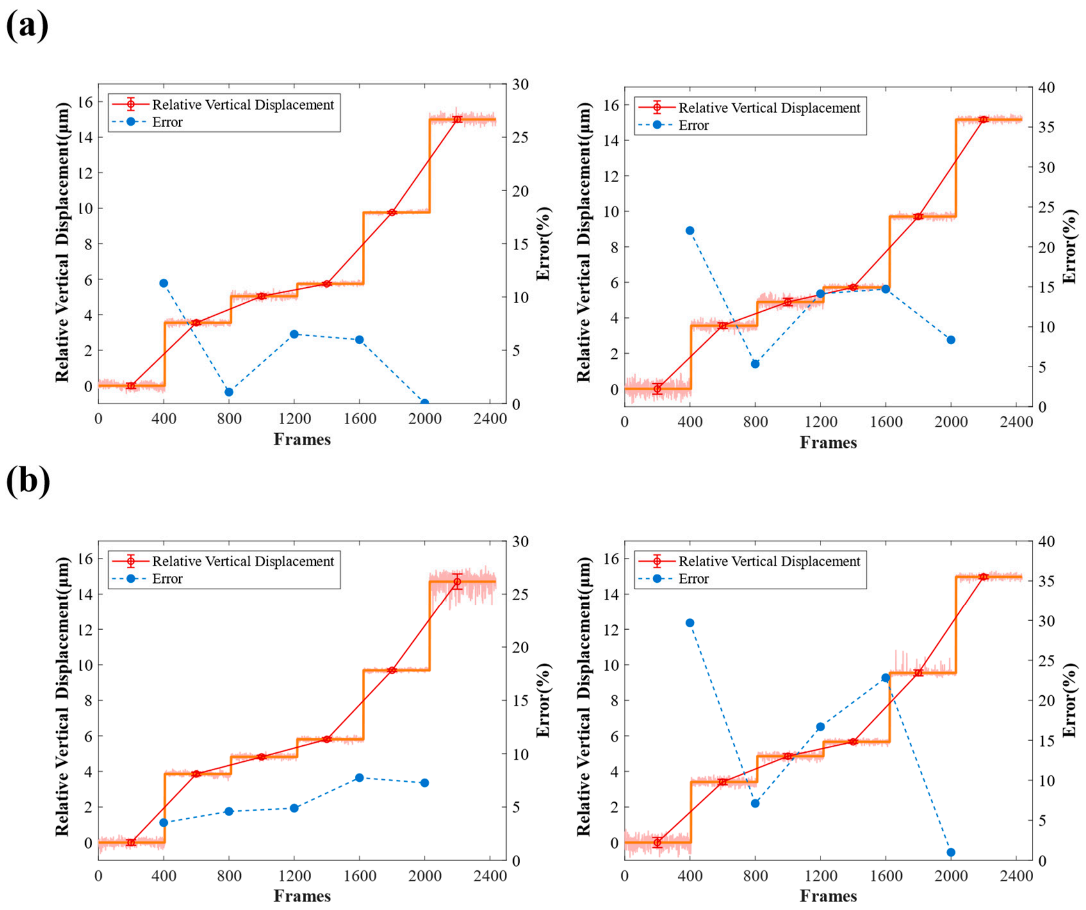

(a)
(b)
Relative Vertical Displacement
Error
Relative Vertical Displacement(μm)
Error(%)
Frames

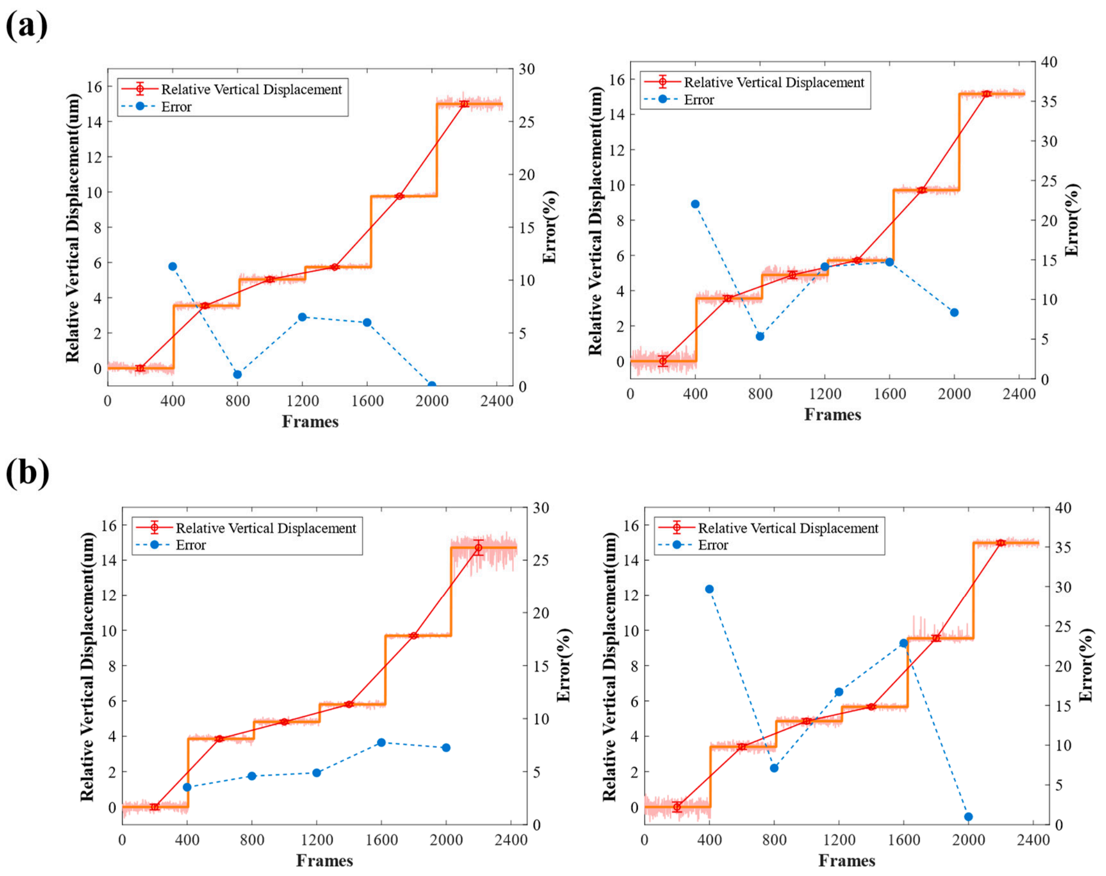

Figure S3 Comparison results of DLA34; (a) Real-time tracking and the tracking data distribution along the z-axis of the big particle and small particle for CenterXFNet-DLA34, respectively. (b) The tracking data distribution of big particles along the z-axis of the big particle and small particle for CenterNet-DLA34, respectively.

Supplementary Figure S4

(a)

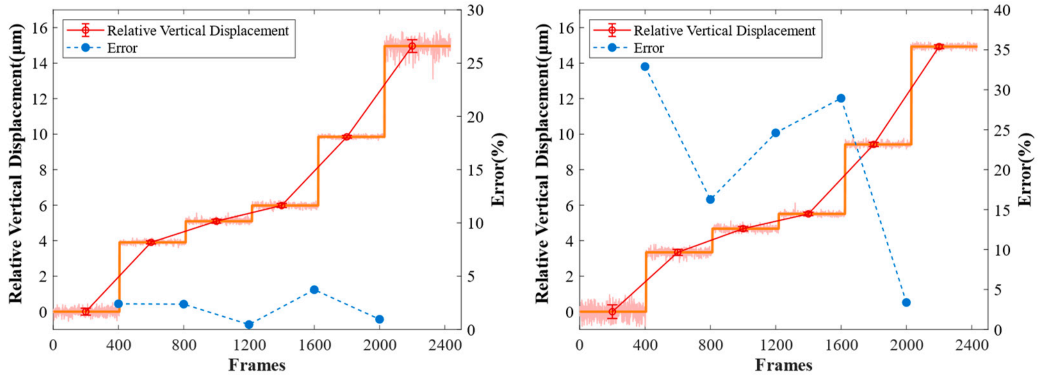

Relative Vertical Displacement
Error
Relative Vertical Displacement(μm)
Error(%)
Frames
0
400
800
1200
1600
2000
2400

(b)

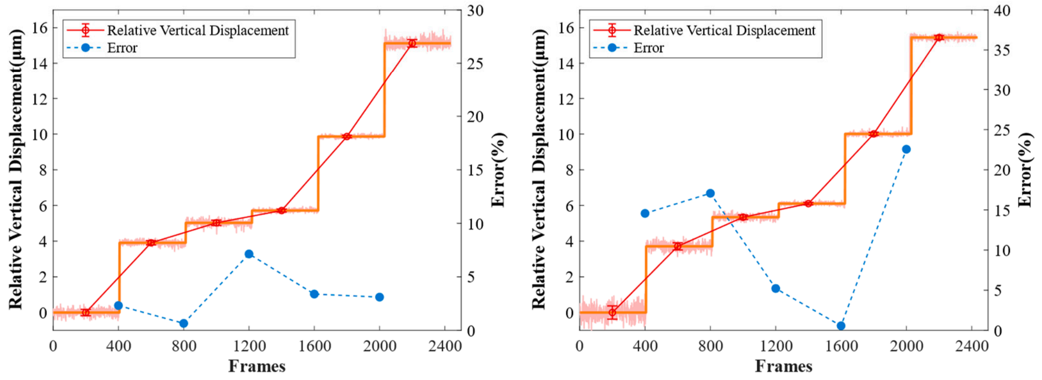

Relative Vertical Displacement
Error
Relative Vertical Displacement(μm)
Error(%)
Frames
0
400
800
1200
1600
2000
2400

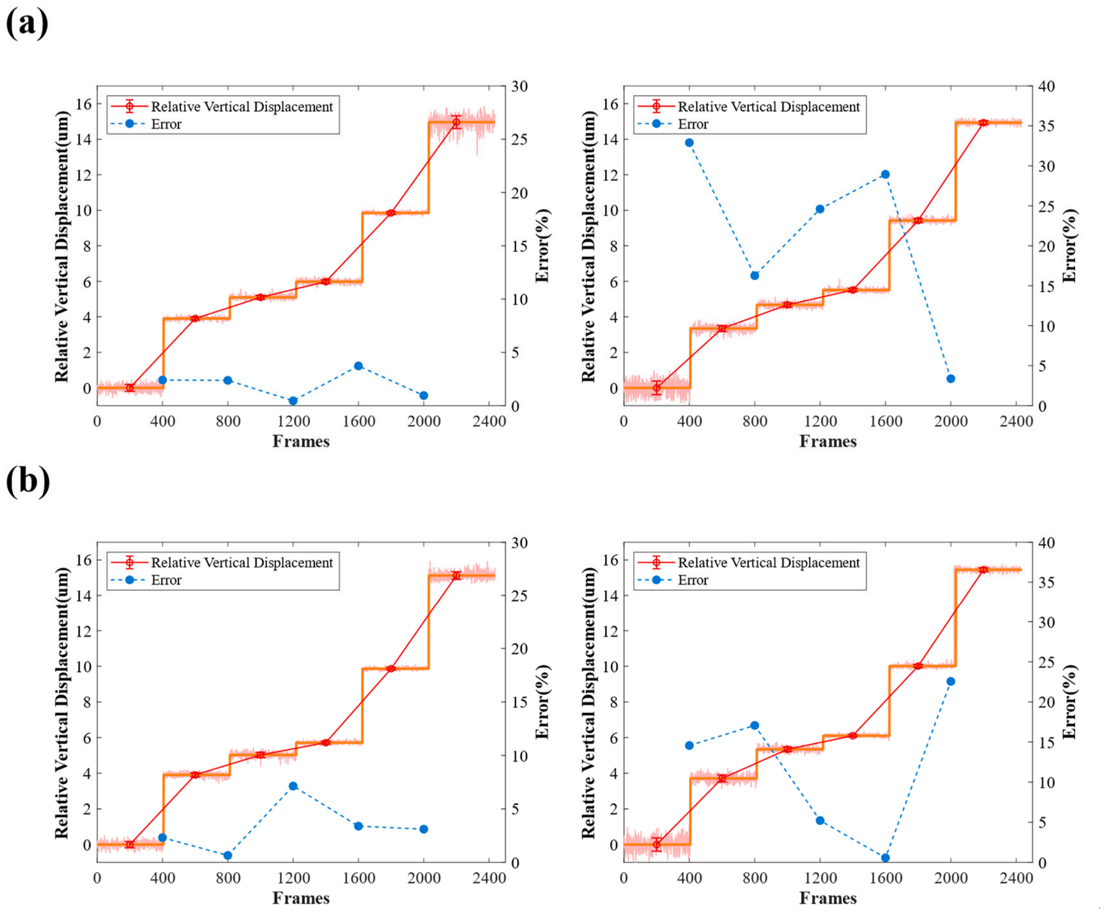

Figure S4 Comparison results of ResNet101 (a) Real-time tracking and the tracking data distribution along the z-axis of the big particle and small particle for CenterXFNet-ResNet101, respectively. (b) The tracking data distribution along the z-axis of the big particle and small particle for CenterNet-ResNet101, respectively.
